# The role of tau in the pathological process and clinical expression of Huntington’s disease

**DOI:** 10.1093/brain/awv107

**Published:** 2015-05-07

**Authors:** Romina Vuono, Sophie Winder-Rhodes, Rohan de Silva, Giulia Cisbani, Janelle Drouin-Ouellet, REGISTRY Investigators of the European Huntington’s Disease Network, Maria G. Spillantini, Francesca Cicchetti, Roger A. Barker

**Affiliations:** 1 John van Geest Cambridge Centre for Brain Repair, Department of Clinical Neuroscience, University of Cambridge, Cambridge, UK; 2 Institute of Psychiatry, Psychology and Neuroscience, King’s College London, London, UK; 3 Reta Lila Weston Institute, UCL Institute of Neurology, London, UK; 4 Centre de Recherche du CHU de Québec (CHUQ), Axe Neuroscience and Département de Psychiatrie et Neurosciences, Québec, QC, Canada; 5 Université Laval, Québec, QC, Canada

**Keywords:** Huntington’s disease, tau, neurofibrillary tangles, dementia, neuropathology

## Abstract

Huntington’s disease is a neurodegenerative disorder caused by an abnormal CAG repeat expansion within exon 1 of the huntingtin gene *HTT*. While several genetic modifiers, distinct from the Huntington’s disease locus itself, have been identified as being linked to the clinical expression and progression of Huntington’s disease, the exact molecular mechanisms driving its pathogenic cascade and clinical features, especially the dementia, are not fully understood. Recently the microtubule associated protein tau, MAPT, which is associated with several neurodegenerative disorders, has been implicated in Huntington’s disease. We explored this association in more detail at the neuropathological, genetic and clinical level. We first investigated tau pathology by looking for the presence of hyperphosphorylated tau aggregates, co-localization of tau with mutant HTT and its oligomeric intermediates in post-mortem brain samples from patients with Huntington’s disease (*n* = 16) compared to cases with a known tauopathy and healthy controls. Next, we undertook a genotype–phenotype analysis of a large cohort of patients with Huntington’s disease (*n* = 960) with a particular focus on cognitive decline. We report not only on the tau pathology in the Huntington’s disease brain but also the association between genetic variation in *tau* gene and the clinical expression and progression of the disease. We found extensive pathological inclusions containing abnormally phosphorylated tau protein that co-localized in some instances with mutant HTT. We confirmed this related to the disease process rather than age, by showing it is also present in two patients with young-onset Huntington’s disease (26 and 40 years old at death). In addition we demonstrate that tau oligomers (suggested to be the most likely neurotoxic tau entity) are present in the Huntington’s disease brains. Finally we highlight the clinical significance of this pathology by demonstrating that the *MAPT* haplotypes affect the rate of cognitive decline in a large cohort of patients with Huntington’s disease. Our findings therefore highlight a novel important role of tau in the pathogenic process and clinical expression of Huntington’s disease, which in turn opens up new therapeutic avenues for this incurable condition.

## Abstract (precis)

Tau has recently been implicated in Huntington’s disease, but the nature of its involvement is unclear. Vuono *et al.* reveal tau oligomers and hyperphosphorylated tau aggregates in post-mortem Huntington’s disease brains, including those from young-onset cases. Genotype-phenotype analysis of a large patient cohort shows that tau haplotypes influence cognitive decline.

## Graphical abstract

**Figure:**
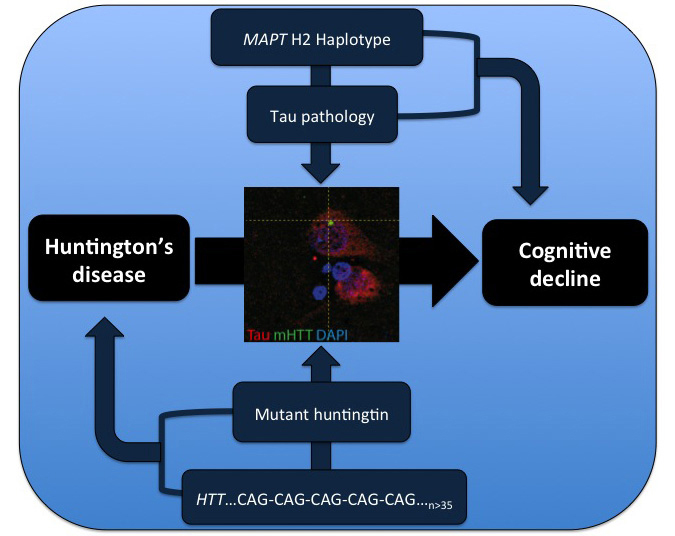
Tau has recently been implicated in Huntington’s disease, but the nature of its involvement is unclear. Vuono *et al.* reveal tau oligomers and hyperphosphorylated tau aggregates in post-mortem Huntington’s disease brains, including those from young-onset cases. Genotype-phenotype analysis of a large patient cohort shows that tau haplotypes influence cognitive decline.

## Introduction

Huntington’s disease is an autosomal dominant neurodegenerative disorder characterized by motor, psychiatric and cognitive deficits. Cognitive impairment appears early in the disease course, often leading to dementia which profoundly impacts on quality of life (Ready *et al.*, 2008; Novak and Tabrizi, 2011). Huntington’s disease is caused by an abnormal CAG repeat expansion within exon 1 of the human huntingtin gene (*HTT*) (Gusella *et al.*, 1983) encoding the huntingtin (HTT) protein. The expansion results in a mutant HTT protein, which forms oligomers and globular intermediates that give rise to aggregates associated with widespread neuronal dysfunction and neurodegeneration, particularly within the cerebral cortex and striatum. Although there is a correlation between CAG repeat length and age at onset of motor features (The Huntington’s Disease Collaborative Group, 1993; Ranen *et al.*, 1995; Zuccato *et al.*, 2010), patients with Huntington’s disease differ dramatically in their age at onset, initial disease manifestations and progression, despite similar CAG repeat length. Several studies have identified a large set of possible genes (Zuccato *et al.*, 2010), distinct from the Huntington’s disease locus itself, that could modify the disease manifestation and progression but their relevance remains unclear. As such there is a need to find other genes and/or genetic variants, which will provide novel clues to the complex pathogenesis of Huntington’s disease. One such candidate is the microtubule-associated protein tau (*MAPT*) gene.

The *MAPT* gene is localized on chromosome 17q21 and is represented by two haplotype clades, the more common H1 haplotype and the rarer H2 haplotype, which result from a large (∼970 kb) chromosomal inversion (Stefansson *et al.*, 2005). The H1 haplotype has been consistently shown to be a strong genetic risk factor for progressive supranuclear palsy, corticobasal degeneration (Baker *et al.*, 1999; Houlden *et al.*, 2001) and we have previously shown that it is also associated with the earlier development of dementia in Parkinson’s disease (Goris *et al.*, 2007; Williams-Gray *et al.*, 2009).

The adult human brain expresses six isoforms of the tau (MAPT) protein, which is involved in microtubule assembly, stabilization, neuronal morphogenesis, and axonal transport (Spillantini and Goedert, 2013). Alternative splicing of exon 10 results in isoforms with three (3R) or four (4R) carboxy-terminal microtubule-binding repeat domains expressed in approximately equal amount in the CNS of healthy adults (Goedert *et al.*, 1989). Mutations and common genetic variants of the *MAPT* gene have been associated with the development of tauopathies as well as to imbalances in the 4R:3R tau isoform ratio which may contribute to tau hyperphosphorylation, aggregation and pathology (Anfossi *et al.*, 2011; Spillantini and Goedert, 2013).

Recently it has been shown that there is an increase in the 4R:3R tau isoform ratio, with nuclear rod-like tau deposits (TNRs) in Huntington’s disease post-mortem brain samples as well as an attenuated motor phenotype in mutant HTT transgenic mice with genetic tau reduction (Fernández-Nogales *et al.*, 2014). Subsequently, it has been shown that, in transgenic Huntington’s disease mouse models, mutant HTT alters tau phosphorylation (Blum *et al.*, 2015; Gratuze *et al.*, 2015) which is known to be a characteristic of insoluble paired helical filaments that form neurofibrillary tangles (Grundke-Iqbal *et al.*, 1986; Kosik *et al.*, 1986; Wood *et al.*, 1986; Goedert *et al.*, 1988, 1992). Indeed, neurofibrillary tangles have been detected in Huntington’s disease brains (Jellinger, 1998) although these findings were attributed to co-incidental Alzheimer’s disease (Davis *et al.*, 2014).

Given all this, there is still debate surrounding the question of whether tau aggregates and their level of phosphorylation affect the expression and/or progression of Huntington’s disease (Hernández and Avila, 2008; Caparros-Lefebvre *et al.*, 2009). In this study, we set out to answer this question by first of all ascertaining whether there are hyperphosphorylated tau aggregates in cortical and striatal tissues from patients with Huntington’s disease, with comparison to cases with a known tauopathy (Alzheimer’s disease, progressive supranuclear palsy, Pick’s disease and corticobasal degeneration) and healthy control subjects. We then sought to confirm the presence of pathological tau in patients with young-onset Huntington’s disease (26 and 40 years old at death) to show that the findings were linked to the disease and not old age. Finally we investigated the relationship between *MAPT* haplotypes and the clinical progression of the disease, with a focus on cognitive decline, by performing a genotype–phenotype analysis in a large cohort of individuals with Huntington’s disease.

## Materials and methods

### Ethics statement

The study was approved by the Local Research Ethics Committee and the other sites of the European Huntington Disease Network (EHDN) REGISTRY project (Orth *et al.*, 2011). The participants and/or the next of kin gave informed written consent for the use of genetic material and brain tissue for research according to International Conference on Harmonisation of Technical Requirements for Registration of Pharmaceuticals for Human Use-Good Clinical Practice (ICH-GCP) guidelines (http://www.ich.org/LOB/media/MEDIA482.pdf) and the Declaration of Helsinki.

### Subjects

Human genetic material, clinical information and CAG repeat length data were obtained from the European Huntington’s Disease Network (EHDN) REGISTRY (Orth *et al.*, 2011; http://www.euro-hd.net/html/registry). Data were available from 960 patients who had a clinical and genetically confirmed diagnosis of Huntington’s disease. Age at onset was defined as the age at which their first Huntington’s disease features appeared as judged by a trained neurologist either from the neurological examination or (more frequently) from the patient history as recorded in REGISTRY. Motor, functional and cognitive features were scored at visits ∼1 year apart using the Unified Huntington Disease Rating Scale (UHDRS’99; Huntington Study Group, 1996). Cognitive assessments included tests of verbal fluency, the digit-symbol modality test and the Stroop test (word, colour and interference subtests), all of which are known to be sensitive to the disease process in Huntington’s disease (Ho *et al.*, 2002).

The Cambridge Brain Bank provided anonymous post-mortem brain samples (fresh-frozen and paraffin-embedded tissues) from Huntington’s disease and sporadic tauopathy cases as well as healthy age-matched controls known not to have any neurological or psychiatric disorder (Table 1). Cortical and striatal tissue was available for all cases. Clinical data were retrospectively obtained from the clinical charts available at the Brain Bank and are summarized in Table 1. All the Huntington’s disease cases and healthy controls were assessed by a neuropathologist to ensure they were free of any Alzheimer’s disease pathology according to standard criteria based on tau pathology and amyloid-β deposition (Braak and Braak, 1991).

**Table 1:** Huntington’s disease, tauopathy and control cases

Cases	Gender	Age (years)	Grade (0–4)	CAG_n_ Repeats	CAG_n_ Repeats
Huntington’s disease					
HD1	M	71	1	19	46
HD2	M	61	2	17	46
HD3	F	78	2	17	43
HD4	M	57	3	19	47
HD5	F	64	4	19	45
HD6	M	60	1	23	46
HD7	M	71	1	17	44
HD8	F	61	2	19	46
HD9	M	66	3	17	45
HD10	M	77	3	24	43
HD11	M	77	3	19	43
HD12	M	73	3	21	45
HD13	M	68	4	19	45
HD14	M	58	4	20	48
HD15	M	40	4	18	51
HD16	F	26	4	22	70
	F:M 4:12	Average 63 ± 13.80			
Sporadic tauopathies					
AD	F	78		18	18
PSP	M	75		20	20
PiD	F	68		19	24
CBD	M	79		28	33
	F:M 2:2	Average 75 ± 4.97			
Controls					
CTL1	M	67	0	20	
CTL2	M	72	0	21	
CTL3	M	69	0	20	
CTL4	M	80	0	20	
CTL5	F	83	0	19	
	F:M 1:4	Average 74.2 ± 6.98			

AD = Alzheimer's disease; CBD = corticobasal degeneration; PiD = Pick's disease; PSP = progressive supranuclear palsy.

The pathological severity of Huntington’s disease was scored according to the Vonsattel grading system (Vonsattel *et al.*, 1985; Vonsattel and DiFiglia, 1998).

### Immunohistochemistry

Immunohistochemistry was performed on 10 -μm thick paraffin-embedded sections from cortical and striatal Huntington’s disease brains using monoclonal mouse anti-HTT and anti-tau antibodies and following standard protocols. Deparaffinized and rehydrated tissue sections were incubated overnight at 4°C with the primary antibodies listed in Supplementary Table 1.

Sections to be stained with both RD3 and RD4 antibodies, specific for 3R- and 4R-tau isoforms, respectively (de Silva *et al.*, 2003), required pretreatment (antigen retrieval) by microwaving in 10 mM sodium citrate buffer (pH 6, 0.05% Tween 20) for 20 min. The labelling was visualized with the ABC Elite Vectastain® Kit (Vector laboratories). Briefly, sections were incubated for 2 h at room temperature with the biotinylated secondary antibody (1:500) and, following washes in PBS, horseradish peroxidase Avidin-D was added for 1 h at room temperature and visualized with 3-3’diaminobenzidine as the chromogen.

Controls omitting the primary reagent were included in all the experiments and were consistently negative for any staining. Pre-adsorption controls with 3R- and 4R-tau isoforms were also carried out and found to be negative. Furthermore, the immunohistochemistry performed on healthy control brain sections using all the tau antibodies (Supplementary Table 1) was also negative (Supplementary Fig. 1).

### Immunofluorescence

The experimental procedure was based on previously published protocols (Cisbani *et al.*, 2013; Cicchetti *et al.*, 2014). In brief, prior to immunostaining, sections were deparaffinized in Citrisolv™ (Fisherbrand), rehydrated and incubated overnight at room temperature in blocking solutions containing the primary antibodies listed in Supplementary Table 1. After KPBS washes, sections were incubated with appropriate secondary antibodies in a blocking solution for 2 h at room temperature (Goat anti-mouse Alexa Fluor® 488, 1:750; Goat anti-rabbit Alexa Fluor® 546, 1:500; Goat anti-chicken Alexa Fluor® 647, 1:500; all Life Technologies).

Following DAPI incubation (2 mg/ml, Molecular Probes), sections were washed and coverslipped with Fluoromount-G™ (SouthernBiotech). Confocal laser scanning microscopy was performed using an Olympus FV500 confocal laser-scanning microscope. Images were acquired by sequential scanning and optimized by a two-frame Kalman filter and analysed using acquisition software from Olympus (Fluoview SV500 imaging software 4.3, Olympus) and ImageJ (NIH).

### Immunoblotting

Preparation of soluble and insoluble tau fractions from Huntington’s disease, tauopathy and control brains was adapted from previously published methods (Goedert *et al.*, 1989; Goedert and Jakes, 1990; Hanger *et al.*, 2002). Protein samples were dephosphorylated using λ-protein phosphatase (NEB) at a final concentration of 20 U/μl for 3 h at 30°C. Dephosphorylation reactions were stopped by the addition of LDS sample buffer (Life Technologies) followed by heating at 70°C for 10 min. Samples were centrifuged at 10 000 *g* (av) before separation on NuPAGE 4–12% Bis-Tris gels (Life Technologies) alongside a recombinant tau protein ladder (rPeptide). Proteins were transferred to a nitrocellulose membrane and probed with the phosphorylation-dependent anti-tau mouse monoclonal antibody AT8 (1:1000, ThermoScientific) and a rabbit polyclonal antiserum anti-total tau (Dako, 1:20 000). Blots were visualized and quantified using an Odyssey Infrared imaging system (LI-COR Biosciences).

### RNA isolation and quantitative RT-PCR of tau isoform mRNAs

Total RNA was extracted from the cortical and striatal Huntington’s disease and control brains (200–300 mg) using the RNeasy® Lipid Tissue Midi kit according to the manufacturer’s protocol (Qiagen). The RT-PCR was performed on 500 ng of RNA in a volume of 50 µl by using the Qiagen® Onestep RT-PCR according to standard procedures. The 4R and 3R *MAPT* transcripts were isolated using specific oligonucleotides (ForE9 5′-CTCCAAAATCAGGGGATCGC-3′, RevE12 5′-TTTTTATTTCCTCCGCCAG-3′) (Sigma) as described in Anfossi *et al.* (2011). PCR products were separated by electrophoresis in 1% (w/v) agarose gel. The intensity of the bands was measured and the percentage of products corresponding to the 4R and 3R *MAPT* transcripts was calculated. For all quantitative experiments, RT-PCR reactions were carried out in three independent experiments. Statistical significance was ***P < 0.05, two-tailed *t*-test.

### Genetics

Single nucleotide polymorphism (SNP) genotyping for rs9468, tagging the *MAPT* H1 versus H2 haplotype, was performed using the TaqMan® allelic discrimination assay on an HT7900 sequence detection system (Applied Biosystem), according to the manufacturer’s instructions. Genotyping success rates were >96%. There were no inconsistencies amongst 192 samples genotyped in duplicate.

The CAG size in the *HTT* gene was determined for all the Huntington’s disease and control brain samples by DNA sequencing at the Laragen's Sanger Sequencing Services (CA) using the GeneMapper Software-Applied Biosystem. The results are reported in Table 1.

### Statistical analysis

A χ^2^ test was used to compare the allele frequency of each variant with that expected for a population in Hardy-Weinberg equilibrium. Fisher’s exact test was used to compare H1/H1, H1/H2 and H2/H2 genotype distribution. Only genotyped individuals for whom a complete data set was available for at least two visits, a minimum of 1 year apart, were included in the analysis. These participants were divided into two groups on the basis of their *MAPT* genotype (H1 homozygotes and H2 carriers) as described previously (Goris *et al.*, 2007; Williams-Gray *et al.*, 2009). Baseline demographic and clinical data were compared between groups using two-tailed *t*-tests (two groups) and ANOVAs (more than two groups).

The primary outcome of interest was the annual change in cognitive performance based on a ‘composite cognitive score’, a sum of individual scores in the Verbal Fluency, the Symbol Digit and all parts of the Stroop test (colour, word and interference). Rate of change (points/year) was calculated by subtracting cognitive score at the first assessment from the score at the last follow-up assessment (or most complete data set) divided by the time between these assessments in years, as we have done previously (Goris *et al.*, 2007; Williams-Gray *et al.*, 2009). Because the summed cognitive score will be heavily weighted by inclusion of the three Stroop assessments, change in performance was also calculated for Verbal Fluency, Symbol Digit tests and Stroop (colour, word and interference) sub-tests individually. Our secondary outcome measure was the rate of change in motor decline, calculated using the total motor score from the UHDRS’99 collected at the same visits as described above using an equivalent formula.

Outliers were identified and the data were winsorized using Tukey’s Hinge estimates. The difference in the distributions of rate of cognitive and motor changes per year between genetic groups was first evaluated non-parametrically using Mann-Whitney U-tests (two subgroups). Following this, a multivariate linear regression was used to compare genetic groups with the addition of relevant covariates including CAG repeat length, gender, age at first assessment and years since motor onset at first assessment. Correlations with continuous variables (CAG repeat length) were explored non-parametrically using Kendall’s tau_b_ tests (two-tailed). All analyses were performed using SPSS version 16 and the threshold for statistical significance was *P* < 0.05.

## Results

### Hyperphosphorylated AT8-positive tau aggregates in cortical and striatal Huntington’s disease tissues

**Figure 1:**
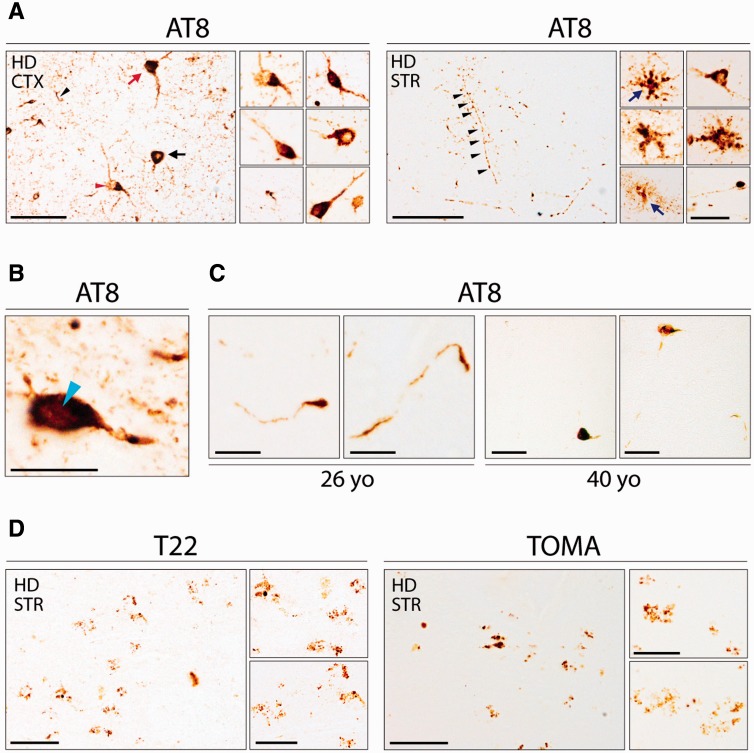
**Tau pathology in Huntington’s disease brains**. (**A**) Immunohistochemical detection of pathological tau aggregates in cortical (CTX) and striatal (STR) Huntington’s disease tissue [HD8, female, 61 years old (yo)] using the AT8 antibody against phosphorylated tau. Neuronal inclusions including ring-like perinuclear (black arrow), flame shaped (red arrow), and globular (red arrowhead) morphologies were observed. Tufted astrocytes and astrocytic plaques (blue arrows) numerous dots and neuropil threads (black arrowhead) were also seen. Scale bars = 50 μm, insets = 20 μm. (**B**) Nuclear rod-like tau deposits positive for AT8-immunoreactivity in Huntington’s disease brains (light blue arrowhead). Scale bar = 20 µm. (**C**) Phosphorylated tau aggregates detected by immunohistochemistry using AT8 in both cases of patients with young onset Huntington’s disease (26 and 40 years old). Scale bars = 10 µm (26-years-old) and 50 µm (40-years-old). (**D**) Immunohistochemistry using TOMA and T22 antibodies in striatal Huntington’s disease tissue (HD8, female, 61-years-old) showing oligomeric tau inclusions. Scale bars = 100 μm, insets = 10 μm.

In an effort to assess tau phosphorylation and pathology in the Huntington’s disease brain, immunohistochemical analyses using the AT8 monoclonal antibody against abnormally phosphorylated tau were performed. Several AT8-positive neuronal inclusions, including ring-like perinuclear, flame-shaped and globular inclusions (Yoshida, 2006; Spillantini and Goedert, 2013) were found in the Huntington’s disease cortex and striatum (Fig. 1A). In particular, the insular cortex contained the greatest density of hyperphosphorylated tau aggregates, which were distributed throughout all the cellular layers (primarily layers II to VI) with the nucleus accumbens and inferior/anterior putamen being the site of greatest tau pathology in the striatum.

The specificity of the AT8 staining was confirmed in known tauopathy cases (Supplementary Fig. 2). In addition, AT8-positive tufted astrocytes, astrocytic plaques, numerous neuropil threads, dots (Fig. 1A) and nuclear rod-like tau deposits (Fernández-Nogales *et al.*, 2014) were also detected among the AT8-positive inclusions (Fig. 1B). Importantly, AT8-positive tau aggregates were also present in both the younger Huntington’s disease cases (26 and 40 years old at death) (Fig. 1C).

### Oligomeric tau inclusions in striatal Huntington’s disease tissues

Having found abnormal tau phosphorylation in both cortical and striatal regions of Huntington’s brains, we next looked for the presence of tau oligomers by using T22 and TOMA antibodies, which are specific for oligomeric tau intermediates (Gerson and Kayed, 2013; Castillo-Caranza *et al.*, 2014). We observed distinct tau granular T22 and TOMA staining in the striatum and in particular within the putamen but not in the cortex of Huntington’s disease brains (Fig. 1D). The specificity of TOMA staining was tested in Alzheimer’s disease brains (Supplementary Fig. 3).

### Biochemical characterization of sarkosyl-insoluble and soluble tau in Huntington’s disease

**Figure 2:**
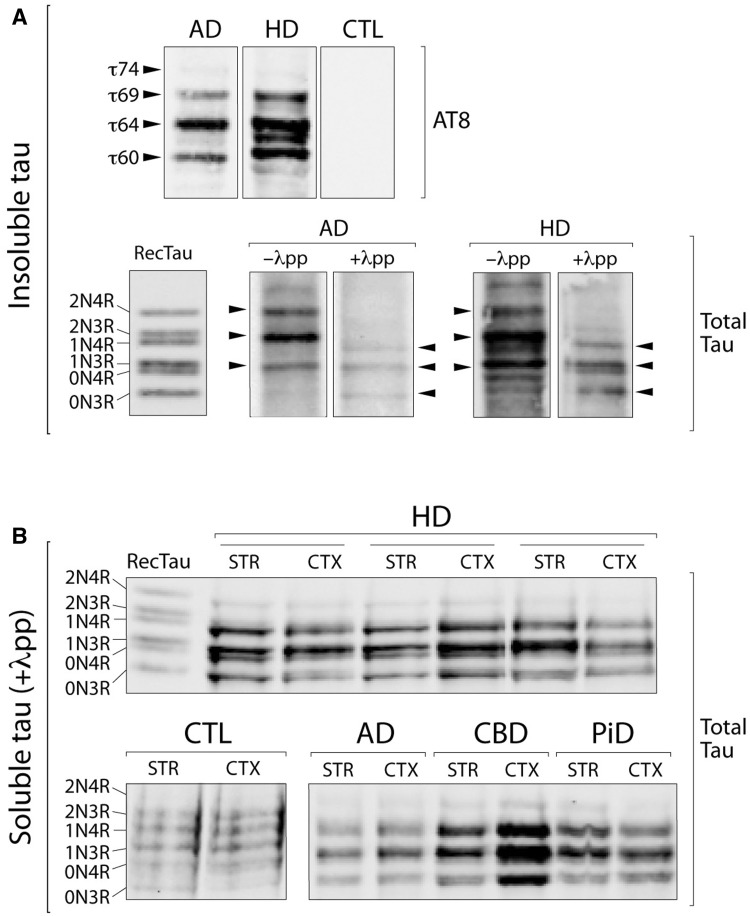
**Biochemical characterization of sarkosyl-insoluble and soluble tau in Huntington’s disease brains**. (**A**) Western blot analysis of the sarkosyl-insoluble tau fraction, before and after dephosphorylation with alkaline phosphatase (λpp), with the AT8 and a total tau antibody in cortical and striatal Huntington’s disease tissues compared to Alzheimer’s disease (AD) cases and healthy controls (CTL). (**B**) Western blot analysis of the soluble tau fraction after dephosphorylation with alkaline phosphatase (λpp) with a total tau antibody in cortical (CTX) and striatal (STR) Huntington’s disease tissues, compared to a range of tauopathies (CBD = corticobasal degeneration; PiD = Pick’s disease) and healthy controls. RecTau molecular weight (kDa): 2N4R (45.9), 2N3R (42.6), 1N4R (42.9), 1N3R (39.7), 0N4R (40.0), 0N3R (36.8).

Our observation of AT8-positive pathology in Huntington’s disease was further supported by western blot analysis with robust staining of sarkosyl-insoluble tau from Huntington’s disease brains (Fig. 2A), which showed a distinct isoform composition and phosphorylation profile compared to other known tauopathies (Sergeant *et al.*, 2005). In addition to the characteristic triplet pattern seen in Alzheimer’s disease with 60, 64 and 69 kDa bands containing 3R- and 4R-tau isoforms, the insoluble tau from Huntington’s disease brains consistently displayed a more complex pattern of weaker bands that confirmed the distinct isoform and/or phosphorylation profiles (Fig. 2A). Dephosphorylation of the sarkosyl-insoluble tau with lambda-phosphatase, followed by western blot analysis with a total tau antibody against all the isoforms, revealed the underlying isoform composition of the insoluble tau with a pattern similar to Alzheimer’s disease samples (Fig. 2A).

To evaluate the level of expression of tau isoforms, the soluble fraction of tau was also extracted from the cortex and striatum of Huntington’s disease brains and compared with control brain samples. All six tau isoforms were expressed in Huntington’s disease, sporadic tauopathy and healthy control brains. However in the Huntington’s disease cases, there was elevated level of the 1N3R and 1N4R isoforms with an electrophoretic pattern closely resembling tauopathy cases, as compared to the healthy controls (Fig. 2B).

### Altered 4R:3R tau isoform ratio in Huntington’s disease brains

We next looked at the 3R- and 4R-tau isoform mRNA levels of expression and, in line with the recent report from Fernández-Nogales *et al.* (2014), found an increased expression of the 4R-tau isoforms transcripts (Supplementary Fig. 4A) and a consequent altered 4R:3R tau transcript ratio in both the cortex (1.261 ± 0.146) and striatum (1.446 ± 0.264) of Huntington’s disease cases versus controls (cortex = 1.037 ± 0.012 and striatum = 1.033 ± 0.059) (Supplementary Fig. 4B).

The presence of 3R- and 4R-tau deposits (Supplementary Fig. 5A and B), as well as nuclear rod-like structures (Supplementary Fig. 5C), was also found in our Huntington’s disease samples. Many of these tau-positive inclusions contained both 3R- and 4R-tau isoforms (Supplementary Fig. 5D). The specificity of the RD3 and RD4 tau antibodies, respectively for the 3R- and 4R-tau isoforms was confirmed in known tauopathy cases (Supplementary Fig. 6).

### Co-localization of mutant HTT and tau in Huntington’s disease brains

We then sought to assess whether there was any co-localization of mutant HTT aggregates and tau deposits and/or pathological phosphorylated aggregates, which would suggest a possible interaction between these two pathological protein species.

**Figure 3:**
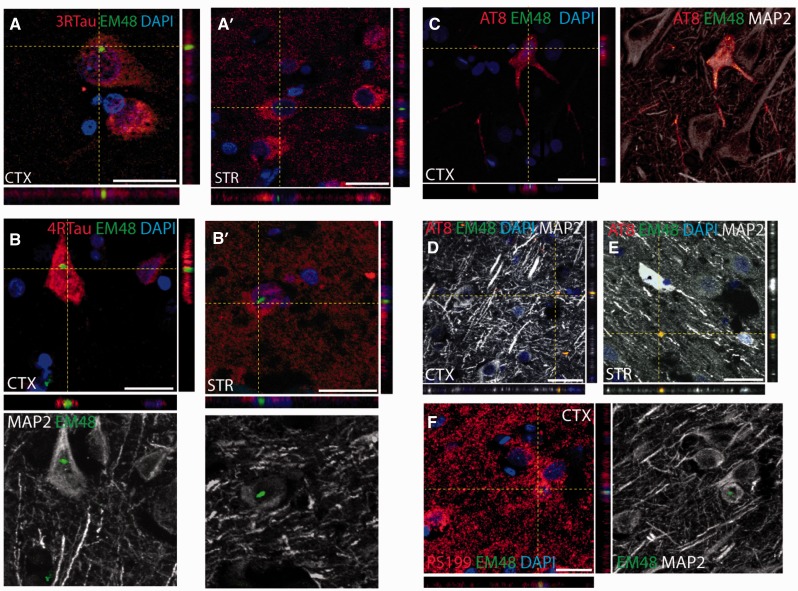
**Mutant huntingtin co-localizes with pathological tau aggregates**. **(A** and **B**) Mutant HTT aggregates labelled with the EM48 antibody (green) detected within cortical and striatal neurons expressing either 3R-tau or 4R-tau (red). Scale bars in **A**, **A’**, **B** = 25 μm; **B’** = 50 μm. (**C–F**) Confocal microscopy images of mutant HTT aggregates labelled with the EM48 antibody (green) within neurons expressing phosphorylated tau (red) as demonstrated using either the AT8 or pS199 antibodies. The neuronal morphology was further confirmed using the neuronal marker MAP2 (grey). Aggregates stained for both EM48 and AT8 were also found in the extracellular matrix of the cortex and striatum of Huntington’s disease cases as shown in **D** and **E**. Scale bars in **C–E** = 30 μm; **F** = 20 μm.

Huntington’s disease cortical and striatal sections were therefore stained with antibodies specific for 3R- and 4R-tau isoforms (RD3 and RD4, respectively), pathologically phosphorylated tau (AT8 and pS199) and mutant HTT (EM48). The results revealed that some of the mutant HTT aggregates co-localized with both the 3R- and 4R-tau deposits (Fig. 3A and B) and the pathological phosphorylated tau aggregates (Fig. 3C–F).

### Effect of *MAPT* H2 haplotype on cognitive decline in patients with Huntington’s disease

Having established clear evidence of tau pathology in the brains of patients with Huntington’s disease, and a possible interaction with mutant HTT aggregates, we then investigated whether *MAPT* haplotypes—known to alter the expression of tau isoforms—had any impact on the development and clinical expression of Huntington’s disease using genotype-phenotype analysis.

A total of 960 Huntington’s disease cases were genotyped for the H1 and H2 haplotypes using the SNP rs9468 and no marker showed evidence of deviation from Hardy-Weinberg equilibrium. Complete genetic and clinical data (including data from all five cognitive tests) for two independent assessments at least a year apart were available for 473 of these individuals who were then included in the analysis. Of these, 60% (*n* = 283) were H1 homozygotes (H1/H1) and 40% (*n* = 190) were H2 carriers (*n* = 161 H1/H2 and *n* = 29 H2/H2) (Table 2).

**Table 2:** Demographic, clinical and genotypic characteristics of the EHDN Huntington’s disease cohort

	H1/H1	H1/H2	H2/H2	F/ChiSq	*P*^a^
*n*	283	161	29		
Age^b^	49.41(12.88)	50.55 (10.98)	48.07 (12.27)	0.727	0.484
CAG repeat length	44.45 (4.74)	43.76 (3.12)	44.86 (5.11)	1.646	0.194
Years since disease onset^b^	7.29 (5.01)	7.26 (4.65)	5.33 (4.03)	2.210	0.111
Interval between visits (years)	1.88 (1.16)	1.97 (1.14)	1.83 (0.99)	0.430	0.691
Gender(F:M)	137:146	83:78	11:18	1.876	0.391
UHDRS Motor score^b^	28.11 (15.71)	28.62 (16.38)	26.41 (20.31)	0.231	0.794
UHDRS Functional score^b^	9.31 (2.94)	9.27 (2.89)	9.52 (3.57)	0.087	0.916
Cognitive score^b^	176.88 (60.19)	178.31 (63.20)	185.76 (79.23)	0.271	0.763

Group means are shown with standard deviations in parenthesis. UHDRS = Unified Huntington's Disease Rating Scale; M = Male; F = Female.

^a^Derived from independent one-way ANOVA or χ^2^ tests for categorical data.

^b^At first visit.

**Figure 4:**
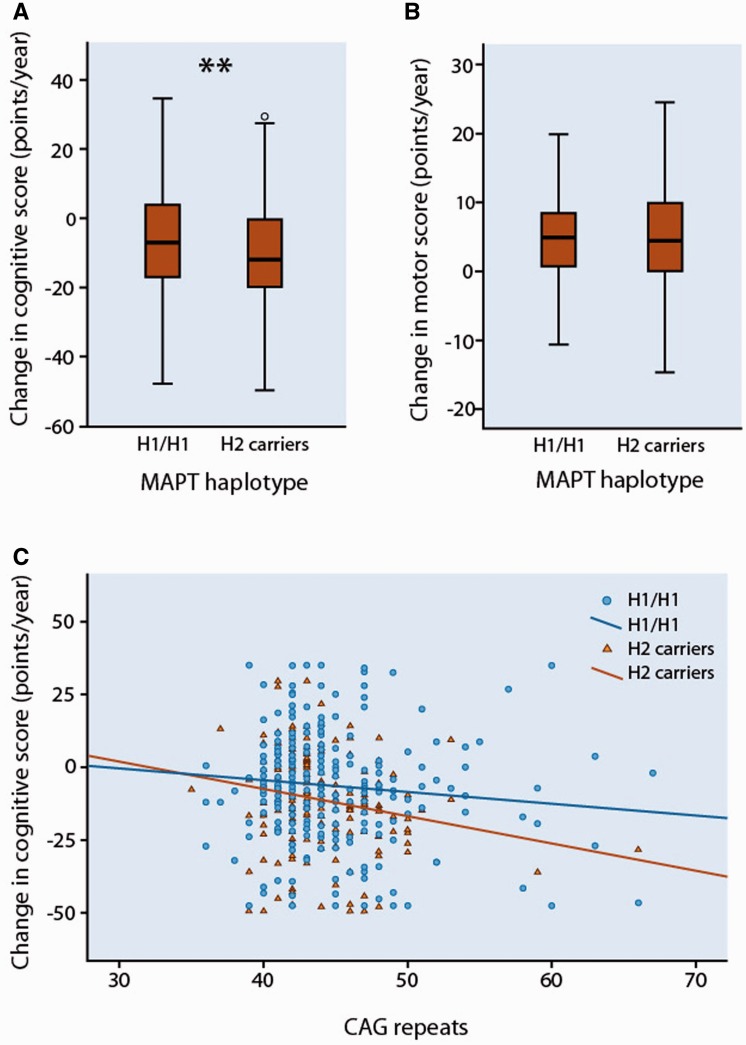
**The effect of *MAPT* haplotypes on cognitive and motor decline in patients with Huntington’s disease**. (**A**) Box and whisker plot showing rates of change in composite cognitive scores in patients with Huntington’s disease grouped by *MAPT* haplotype. Median, interquartile range, and minimum-maximum range are shown. Distributions were compared using Mann–Whitney U-tests. **P < 0.01. (**B**) Box and whisker plot showing rates of change in UHDRS Motor scores in patients with Huntingtons' disease grouped by *MAPT* haplotype. (**C**) Correlation between the CAG repeat length and change in composite cognitive score per year in patients with Huntington’s disease grouped by *MAPT* haplotype. Across all participants (*n* = 473), Kendall’s tau_b_ = −0.098, *P = *0.002. In H1/H1 (squares; *n* = 283), Kendall’s tau_b_ = −0.063, *P = *0.129; in H2 carriers (triangles; *n* = 190); Kendall’s tau_b_ = −0.148, *P = *0.004.

The H1/H1 and H2 carrier groups were demographically matched (Table 2). The mean annual change in the composite cognitive score was −6.31 points [standard deviation (SD) 23.44] in H1 homozygotes compared to −11.31 points (SD 17.66) in the combined group of H2 carriers [−11.61 (SD 17.02) in H1/H2 and −8.75 (SD 11.90) in H2/H2]. Non-parametric comparison confirmed that this corresponded to a significantly higher rate of overall cognitive decline in H2 carriers than in H1 homozygotes (U = 22471, z = −3.029, *P = *0.002) (Fig. 4A). Considering the cognitive subtests individually, there was a significantly higher rate of decline in performance on the Verbal fluency and Stroop Word tests in H2 carriers than H1 homozygotes, with a trend in the same direction for the other tests (Table 3). Multivariate analysis confirmed that the significant association between *MAPT* haplotype and rate of change in composite cognitive score remained even after correction for potential confounding variables (Table 4). Furthermore, the observed effect of *MAPT* haplotype was sustained in a subgroup of 107 patients (62 H1/H1, 45 H2 carriers) who had been followed up over a longer time (mean, 3.52 years; SD, 1.26; U = 915, z = −3.028, *P = *0.002). In contrast, there were no differences associated with *MAPT* haplotype on rate of change in motor performance (Fig. 4B) as measured using the total motor score of the UHDRS; (Tables 3 and 4) and there was no association between *MAPT* haplotype and age at onset (Table 2 and Supplementary Fig. 7).

**Table 3:** Comparisons of rate of change in cognitive and motor scores between H1 homozygotes and H2 carriers with Huntington's disease.

	Median change (points/year)					
	H1/H1	H2 carriers	U	*z*-score	r	*P*
Composite cognitive score	−7.00	−12.00	22471	−3.029	−0.139	0.002
Verbal fluency	0.00	−0.92	23477	−2.339	−0.108	0.019
Symbol digit	−1.09	−1.85	24158	−1.871	−0.086	0.061
Stroop colour	−1.85	−2.40	25157	−1.186	−0.055	0.236
Stroop word	−3.00	−4.62	23572	−2.273	−0.105	0.023
Stroop interference	−1.03	−1.54	25903	−0.674	−0.031	0.500
UHDRS Motor score	4.85	4.43	25649	0.019	0.001	0.985

Non-parametric comparisons of rate of change in cognitive scores (*n* = 473) and Unified Parkinson's Disease Rating Scale (UHDRS) motor scores (*n* = 462) between H1 homozygotes and H2 carriers with Huntington's disease. U = Mann Whitney test statistic, r = effect size; *P* based on two-sided test.

**Table 4:** Multivariate regression showing factors associated with change in cognitive and motor score in Huntington's disease.

Predictor Variable	Change in cognitive score		Change in motor score	
	B coefficient	*P*	B coefficient	*P*
Constant	36.852	0.038	−10.443	0.146
*MAPT* (H1/H1 versus H2 carriers)	−5.291	0.002	0.305	0.647
CAG repeat length	−0.815	0.006	0.320	0.008
Sex	−0.344	0.834	0.168	0.797
Age^a^	−0.111	0.297	0.021	0.620
Years since onset^a^	−0.120	0.493	−0.037	0.591

Multivariate linear regressions with change in composite cognitive score per year (*n* = 473. R^2 ^= 0.039) and change in UHDRS motor score per year (*n* = 462. R^2 ^= 0.030) as the dependent variables.

^a^Predictor variables represent baseline visit measures.

There was a significant effect of CAG repeat length on the rate of cognitive change (Table 4), with a higher number of CAG repeats correlating with an increased rate of cognitive decline across all participants (Kendall’s tau_b_ = −0.098, *P = *0.002). Stratifying the patients according to *MAPT* haplotype revealed that this correlation was only significant in the H2 carriers but not in H1 homozygotes (Fig. 4C). The established negative correlation between CAG repeat length and age at onset was present across all participants (Kendall’s tau_b_ −0.602, *P < *0.001), and in both H2 carrier and H1 homozygote groups when considered separately (Supplementary Fig. 7). There was an association between CAG repeat length and the rate of motor change, with a longer CAG repeat length predicting an increased rate of motor decline (Table 4). Age at assessment did not independently influence the annual rate of change in the composite cognitive or motor score (Table 4).

## Discussion

Tau has been recently reported to be involved in Huntington’s disease (Fernández-Nogales *et al.*, 2014; Blum *et al.*, 2015; Gratuze *et al.*, 2015) although the exact mechanisms as to how this relates to the clinical features of Huntington’s disease, especially the dementia, are not fully understood.

In this study we establish a key involvement of tau in Huntington’s disease. In particular, we confirm and extend the recent findings of Fernández-Nogales *et al.* (2014), by reporting pathological inclusions containing abnormally phosphorylated tau protein that co-localizes with mutant HTT aggregates in the cortex and striatum of human Huntington’s disease brains. Furthermore, tau pathology was clearly evident in all our Huntington’s disease cases and occurred independently of their disease grade, CAG length and age. The co-occurrence of Alzheimer’s disease in the ageing Huntington’s disease brains was controlled by selecting Huntington’s disease cases that were free of any amyloid-β and tau pathology according to standard procedures (Braak and Braak, 1991). Furthermore, our biochemical characterization of the sarkosyl-insoluble tau showed in Huntington’s disease a distinct isoform composition and phosphorylation profile compared to that of Alzheimer’s disease.

Finally we observed tau oligomers, which are suggested to be the most likely neurotoxic tau entity (Gerson and Kayed, 2013; Castillo-Caranza *et al.*, 2014), only in the putamen of these brains, which further reinforces the notion that the presence of tau is a true pathological finding in this condition. However, at this time we are unable to quantify the distribution of tau pathology across the whole Huntington’s disease brain because of the limited brain sites we have examined. Therefore, future work is needed to provide an extensive characterization of pathological tau aggregates in the human Huntington’s disease brain, which will allow a better mapping of tau pathology onto clinical features. Nevertheless, our restricted qualitative analysis on the distribution of abnormally phosphorylated tau aggregates and oligomers did reveal a differential localization in Huntington’s disease brains, with a higher burden of pathological tau in the insular cortex compared to other cortical sites and the nucleus accumbens and inferior/anterior putamen compared to other striatal sites.

In addition, we show for the first time that tau is of clinical significance in Huntington’s disease through our finding that *MAPT* H2 haplotype affects the rate of cognitive decline in a large cohort of patients with Huntington’s disease, with evidence of some interaction with CAG repeat length. Whilst the exact mechanism of this is not known, coupled to our pathological data, this supports a possible molecular interaction between these two proteins, as has been recently suggested by *in vitro* and in *vivo* studies using transgenic animal models of Huntington’s disease (Blum *et al.*, 2015; Gratuze *et al.*, 2015).

Thus the importance of our new findings lies in the unequivocal demonstration of tau pathology in the brains of patients with Huntington’s disease and the effect of *MAPT* H2 haplotype on cognitive decline in a large cohort of patients. This is of particular interest given that the H1 *MAPT* haplotype has consistently been shown to be the risk haplotype for other neurodegenerative disorders including dementia in Parkinson’s disease (Williams-Gray *et al.*, 2009). It is also of note that in progressive supranuclear palsy, corticobasal degeneration and Parkinson’s disease, the H1 haplotype has been associated with increased 4R-tau isoforms while we now report the opposite association in Huntington’s disease. One possible explanation for this may relate to the binding and sequestration of splicing factor-6 (SRSF6/SRp55) by the CAG repeat in mutant *HTT* mRNA (Fernández-Nogales *et al.*, 2014). This may result in a more profound allele-specific effect on spliceosome assembly during transcription of the H2 allele, giving rise to the large increase in 4R-tau mRNA that we observe in Huntington’s disease, especially given that SRSF6 is involved in *MAPT* exon 10 splicing (Yin *et al.*, 2012) and co-localizes with mutant HTT inclusions (Fernández-Nogales *et al.*, 2014).

In summary, we show a novel role of tau in the pathological process and clinical expression of Huntington’s disease. Further studies are needed to elucidate the exact mechanisms by which this occurs, which in turn may open up new therapeutic approaches for treating this currently incurable condition.

## Abbreviations

EHDN: European Huntington Disease Network
UHDRS: Unified Huntington Disease Rating Scale
